# Micro- and Nanostructured Polyaniline for Instant Identification of Metal Ions in Solution

**DOI:** 10.3390/nano9020231

**Published:** 2019-02-08

**Authors:** Agnieszka Michalska, Sebastian Golczak, Krzysztof Langer, Jerzy J. Langer

**Affiliations:** Laboratory for Materials Physicochemistry and Nanotechnology, Faculty of Chemistry, Umultowska 89b; Wielkopolska Centre for Advanced Technologies (WCZT), Umultowska 89c, Adam Mickiewicz University in Poznań, Poznań, Poland; a.michalska@delta-dolsk.pl (A.M.); golczak@amu.edu.pl (S.G.); krzysztof.langer@gmail.com (K.L.)

**Keywords:** polyaniline, nanostructures, alkanethiols, monolayers, nanodetector

## Abstract

The unique properties of nanomaterials enable the creation new analytical devices. Polyaniline (PANI) micro- and nanofiber network, freestanding in the gap between two gold microelectrodes, has been used in a new nanodetector for metal ions in solutions. The gold electrodes were modified with the aid of alkanethiols, forming a self-assembled monolayer (SAM), which is able to block the ion current flow, but also to interact with metal ions when specific functional molecules are incorporated into the layer. The electric field of the trapped metal ions induces change of the electrical conductivity of polyaniline nanofibers in vicinity. A small injected sample (75 μL) of a solution of salt (about 0.5 μg of salt) was enough to induce a reproducible change in the electrical conductivity of polyaniline nano-network, which was registered as a function of time within 10–20 s. The response was proportional to the concentration of ions. It also depends on properties of ions, e.g., the ionic radius, which allows for identification of metal ions by analyzing the parameters of the signal: the retention time (RT), half width (HW), amplitude (A) and integral intensity (INT). The advantage of the new device is the instant responsiveness and easy operation, but also the simple construction based on organic (polymer) technology. The system is “open”—when learned and calibrated adequately, other metal ions can be analyzed. The nanodetector can be used in cases where monitoring of the presence and concentration of metal ions is important.

## 1. Introduction

Advances in nanotechnology have led to the development of new types of efficient and inexpensive chemical and biological sensors and detectors. Those new devices are being designed and fabricated as a new class of analytical equipment, operating in the nano- or/and micro-scale [1]. Nanoscale sensory devices are ideal for various applications, including chemical sensing [2], in vivo medical analysis [3,4] and biological testing [5,6,7,8,9]. The active part of the device can be fabricated from silicon nanowires [10,11], carbon nanotubes [12,13] or polyaniline nanofibers [14,15,16,17].

Polyaniline is a conducting polymer with a uniquely simple doping mechanism, based on acid-base reactions. Furthermore, the change of resistance of nanofibril polyaniline is more than 10 times faster than that of conventional polyaniline thin film [18,19,20,21,22].

**Figure 1:**
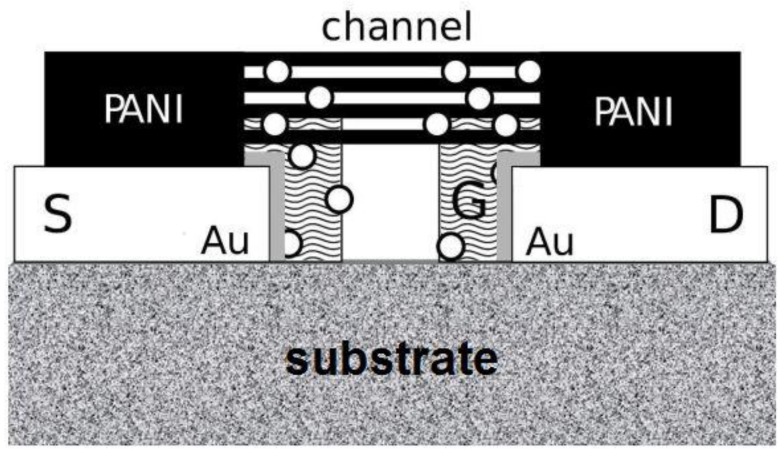
A conceptual scheme of the nanodetector with a mechanism resembling an open-gate ion-sensitive organic field-effect transistor (ISOFET). The “channel” is made of polyaniline nanofibers, and the functional organic monolayer is marked as a “gate” G, which is insulated by a part of the self-assembled monolayer (SAM), formed of alkyl components (here, the thin grey layer is in direct contact with Au electrodes; **o** metal ions).

In this paper, the application of polyaniline micro- and nanofibers in a new nanodetector for metal ions is reported. This is based on Coulomb interactions of ions and polyaniline nanostructures. The mechanism of action is, as a general principle, similar to an open-gate ion-sensitive organic field-effect transistor (ISOFET). The idea behind the device is partially similar to a traditional ion-sensitive field-effect transistor (ISFET), which is the most popular member of a group of chemical field-effect transistors, ChemFET [23,24,25,26]. Detailed information on specific devices and applications one can find in a comprehensive review [27]. However, the mechanism of action, the active materials, the architecture and the construction and technology used are essentially different and innovative (Figure 1).

The advantage of the new device is a simple, miniature construction thanks to the nanotechnology approach, based purely on organic, active components, with no silicon technology. This has been achieved by using a network of polyaniline (PANI) micro- and nanofibers as the detecting element, working similarly to a “channel” of the FET, as well as a specific surface nanomodification of golden electrodes.

**Figure 2:**
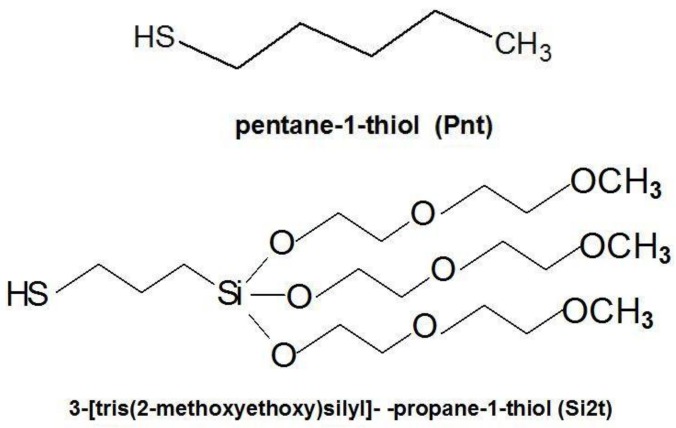
The structure of main alkanethiols used.

**Figure 3:**
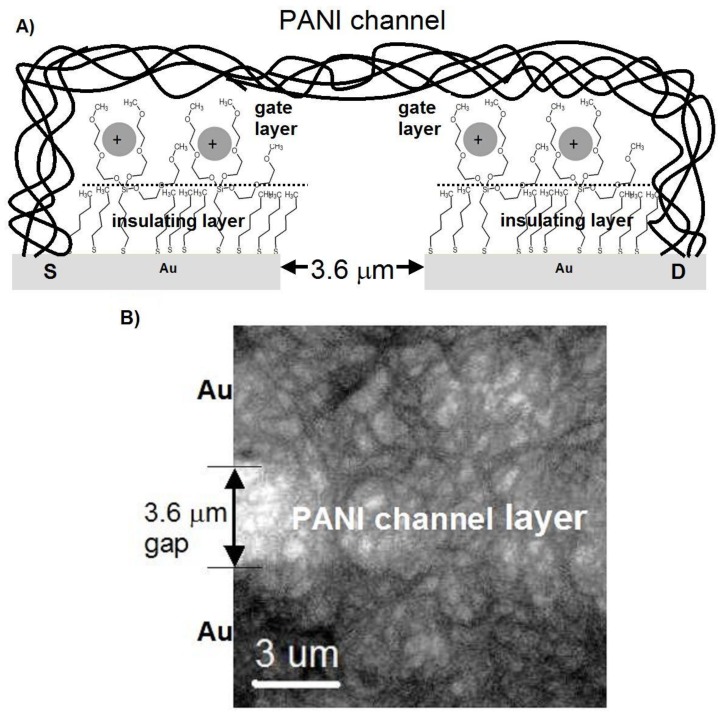
A nanodetector made of polyaniline nanofibers resembles an open-gate ion-sensitive organic field-effect transistor (ISOFET): (**A**) a cross-section (scheme) of the complex SAM Pnt-Si2t (“gate”) deposited onto the Au electrodes (“source” S and “drain” D) in contact with the cations, in vicinity of PANI nanofibrils (“channel”); (**B**) a morphology of the polyaniline network–the “FET channel” (top view, a Scanning Electron Microscope (SEM) micrograph).

In order to significantly limit the ion current, which is necessary to obtain better electrical responses based on the electron current in polyaniline nanofibers, a stable alkanethiol monolayer has been deposited at the surface of the gold electrodes. The top part, the organic layer, due to its specific functional groups, 3-[tris(2-methoxyethoxy)silyl]-propane [28], also plays an active role, similarly to the “gate” of the FET, trapping metal ions (Figure 1, Figure 2 and Figure 3).

**Figure 4:**
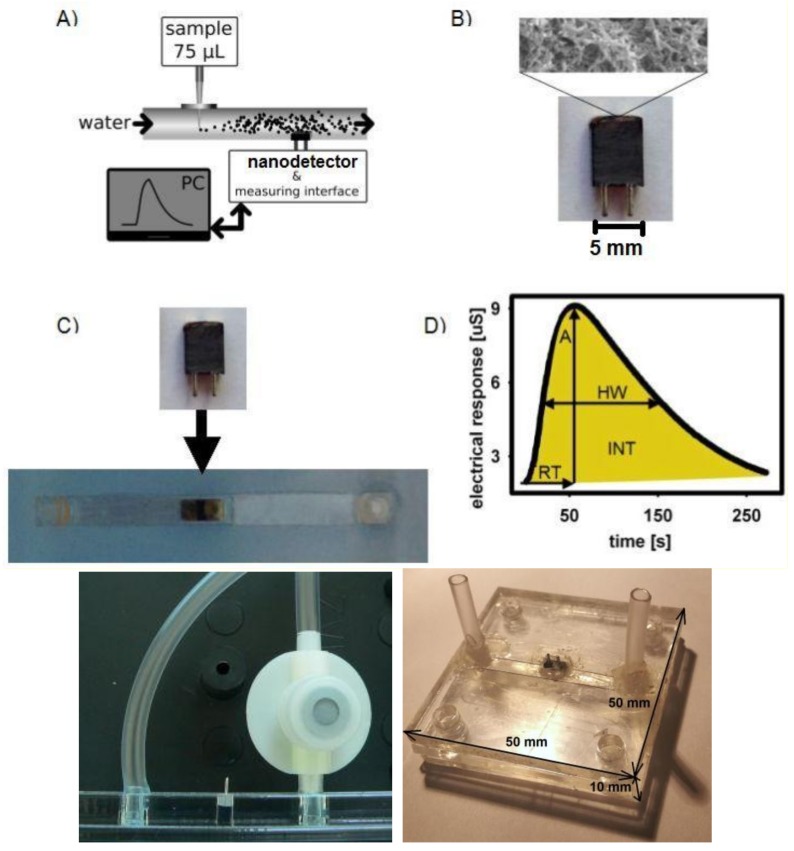
A scheme of the device’s operation in its current version (with the cylindrical flow channel) (**A**); the details of the nanodetector used (**B**); the micro fluidic system with a cuboids channel (**C**) for practical application and implementation (**below**)—designed analogously to that used previously [31,32]; the measured parameters of the signal (**D**): A—the amplitude, RT—the retention time, HW—the half-width, INT—the integral intensity.

This leads to the generation of an electric field close to the polyaniline fibers and to the modification of their electrical conductivity. The last process is very specific, allowing the identification of metal ions based on the dynamic electrical response to sample injection, which is recorded by means of a nanodetector in a continuous flow system (Figure 4A,D).

## 2. Materials and Methods

### 2.1. Materials

Aniline hydrochloride, hydrochloric acid, hexane, potassium hydroxide, benzyl-1-thiol (Bzt), pentane-1-thiol (Pnt) and 3-(trimethoxysilyl)propane-1-thiol (Si1t) were used in the form of products (Sigma-Aldrich Sp. z o.o., Poznań, Poland). 3-[Tris(2-methoxyethoxy)silyl]-propanethiol (Si2t) was prepared in the Laboratory of Supramolecular Chemistry (Faculty of Chemistry, A. Mickiewicz University in Poznan, Poznan, Poland) [28].

### 2.2. Preparation

The main part of the nanodetector is a freestanding polyaniline micro- and nanofibril network, which is in a state of stable electrical contact with the gold electrodes. Firstly, the gold was deposited into a vacuum on a substrate (usually plastic; however, tiny glass plates can also be used). Then, the polyaniline fibrous layer was prepared electrochemically on the gold electrodes at a potential difference of 1 V (versus Ag/AgCl, potentiostat EP20A, ELPAN, Lubawa, Poland). The electrolyte was a 10% aqueous solution of aniline hydrochloride at pH ~ 1 (HCl). The thickness of the PANI layers was controlled with a limited flow of charge. The reaction time was fixed at 300 s. The freshly prepared polyaniline micro- and nanofibril network was washed with distilled water and then dried in air. This is a standard procedure practiced in our laboratory, alongside the characterization of the formed material (polyaniline), as described before [29,30]. Then, the electrodes were treated with a solution of pentane-1-thiol (Pnt; Figure 2) and 3-[tris(2-methoxyethoxy)silyl]propanethiol (Si2t; Figure 2) at a molar ratio of 4:1 to form a self-assembled monolayer (SAM). The monolayer was deposited by immersing gold electrodes (partly covered with PANI nanofibers) into a 3 mM alkanethiols hexane solution for 5 min. Then, the electrodes were washed with hexane and air-dried.

The morphology of the PANI nanonetwork was examined with a Scanning Electron Microscope (SEM–ZEISS EVO 40, Carl Zeiss AG, Oberkochen, Germany). Finally, the nanodetector unit was mounted onto the wall of a channel of the continuous flow system (in this case, a cylinder with a 3 mm diameter).

The device was tested in contact with 0.5 mM aqueous solutions of inorganic salts: NaCl, CaCl_2_, SrCl_2_ and BaCl_2_. The data was collected and registered with a measuring interface supported by a personal computer (PC). The electrical response recorded was proportional to the concentration of cations in the sample injected into the stream of the flowing medium. The measurements were carried out under the following conditions: the medium—distilled water, a flow rate of 6 mL/min and an injected sample of (analyte) 75 μL (tested down to 5 μL). The electrical conductivity was measured in a function of time, with an uncertainty of ±0.1 μS and 0.1 s, using a measuring interface (a conductivity meter, CC-551, ELMETRON, Zabrze, Poland). The results were registered online, by a computer-controlled system.

**Figure A1:**
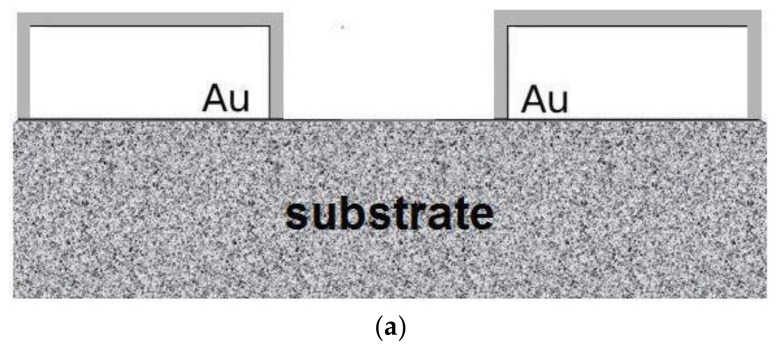

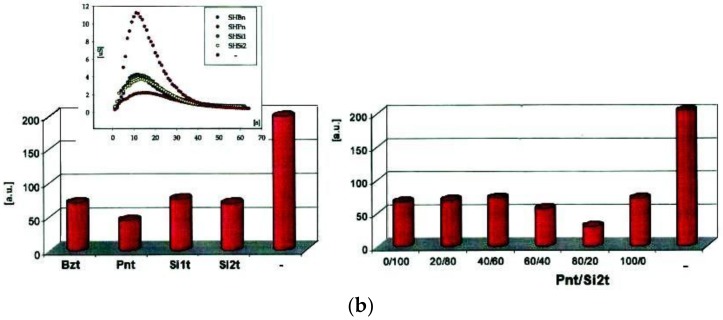
(**a**) A scheme of the system for the preliminary study. The tested blocking monolayer (grey) is formed at the surface of the clean gold electrodes (polyaniline is not deposited in this case) and the current is measured in contact with 0.15 M of NaCl in water; (**b**) the results of the preliminary tests of different blocking layers (with no polyaniline fibers deposited); the integral intensity of the ion current signal (inset), measured in a continuous flow system after the injection of 75 μL of 0.15 M of NaCl in water, for a series of monolayers of different composition: Bzt—benzyl-1-thiol, Pnt—pentane-1-thiol, Si1t—3-(trimethoxysilyl)propane-1-thiol, Si2t—3-[tris(2-methoxyethoxy)silyl]-propane-1-thiol, versus an unmodified gold electrode.

To elaborate the fabrication procedure and to find the right composition, as well as to test the quality and stability of the formed blocking monolayer, a series of preliminary experiments was performed, using clean, gold electrodes (with no polyaniline fibers) and a set of different alkylthiols (Bzt, Pnt, Si1t, Si2t), including their mixtures (e.g., Pnt-Si2t 4:1). The ion current was measured after a sample of 75 μL of an aqueous solution of NaCl in a relatively high concentration (0.15 M) was injected into the same continuous-flow analytical system, though without a fully functional nanodetector. The essential difference was rooted in the absence of polyaniline fibers; thus, the current measured was purely ionic (Appendix A). The results of these tests have been presented in Figure A1b. 

Based on the presented results, a micro fluidic system (Figure 4C), analogous to that used previously [31,32], was designed for future testing of practical applications (Appendix B).

## 3. Results

The device operated in a continuous flow regime, in a testing version with a 3 mm cylindrical flow channel (Figure 4A). For practical applications, a microfluidic lab-on-a-chip-type system was designed (Figure 4C), analogous to our nanobiodetectors, which have been successfully applied to detect vegetative bacteria cells [5,6,31] and spores [31,32]; however, the heart of the device is now substantially different. The main part of the nanodetector for the detection of metal ions in the solution was an active working layer with a thickness of 5–10 μm. It consisted of a network of polyaniline nanofibers with almost uniform fiber diameters of 0.2–0.5 μm (Figure 3B). The fibers were formed electrochemically, directly at the surface of the gold microelectrodes and in between, within a 3–5 μm gap (Figure 3 and Figure 4B). Then, the surface of the gold electrodes was modified to limit the ion current. The best functionality and the best protection against the “parasitic” ion current were achieved with the aid of a monolayer, formed of mixed alkanethiols: pentanethiol (Pnt) and 3-[tris(2-methoxyethoxy)silyl]-propane-1-thiol (Si2t) at a molar ratio of 4:1, Pnt-Si2t (Figure 3A).

This was based on preliminary systematic studies of blocking monolayers of different compositions (Appendix A).

**Figure 5:**
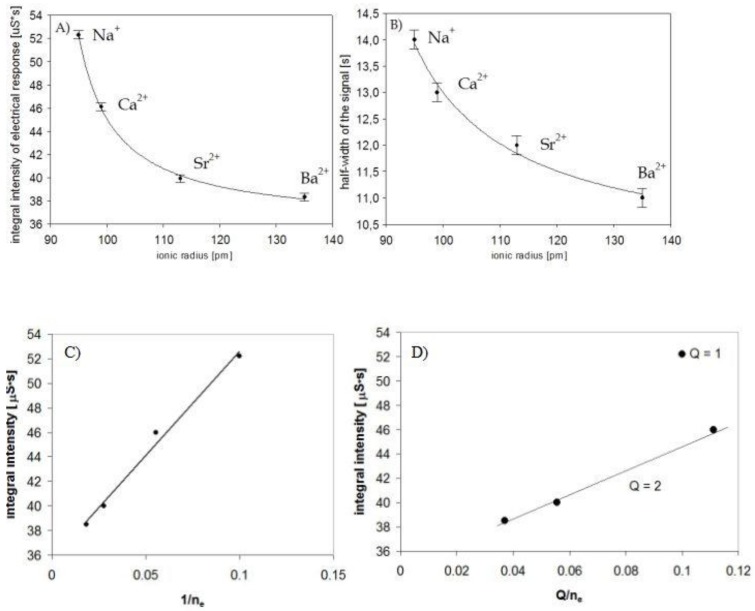
Changes in the signal parameters of the sensor due to the size of the ionic radius of the cations: (**A**) the integral intensity of the electrical response, (**B**) the half-width of the signal, (**C**) the integral intensity of the signals measured for all examined ions versus 1/n_e_, (**D**) the integral intensity of the signals measured for all examined ions versus Q/n_e_, where n_e_ is the number of electrons (n_e_ = Z-Q), Z is the atomic number of a metal and Q is the charge of a metal ion (in our case, Q = +1 or +2, for mono- and divalent ions, respectively).

The mechanism of the action of the new detector resembles a field-effect transistor (FET) [33]. The alkanethiol (Pnt component) monolayer works as an insulator between an FET “source” (S) or “drain” (D) electrodes and the “gate” (G)—Figure 1 and Figure 4. On the other hand, Si2t domains play the role of ion traps, owing to their appropriate structure. Metal ions are trapped by an active centre of complex alkylthiol molecules Si2t (Figure 4) in the vicinity of polyaniline nanofibers. The ions are positively charged; thus, they generate an electric field, which influences the PANI nanofibers (Figure 1 and Figure 4), resulting in a change of their electrical conductivity, owing to a typical field effect. That is why the device resembles an FET. The final result depends on the effective electric field intensity, which is a function of shielding caused by the electrons of a specific metal ion. Consequently, the electrical response of the detector is specific, with respect to the intensity and shape of the signal, registered in a function of time (Figure 4D and Figure 5).

**Figure 6:**
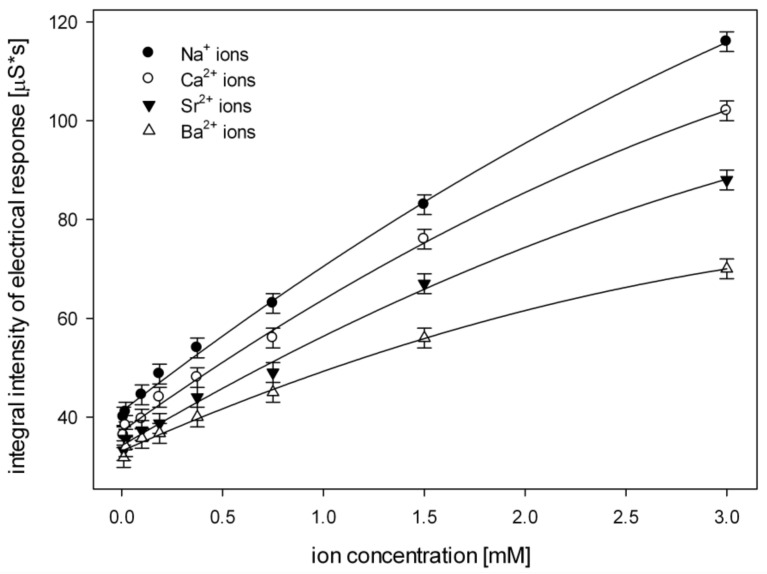
The correlation of the integral intensity of the registered signals and the concentration of Na^+^, Ca^2+^,Sr^2+^ and Ba^2+^ ions.

To test the new nanodetector, a sample of 75 μL of the solution of metal ions in water at a concentration of 0.5 mM is injected into the flowing medium—pure water (Figure 4A) through septa (Figure 4C, bottom left). These are (ionic radii in brackets): Na^+^ (95 pm), Ca^2+^ (99 pm), Sr^2+^ (113 pm), Ba^2+^ (135 pm). The concentration of 0.5 mM was chosen for testing because of its possible biomedical applications; however, a wide range of working conditions can be applied: the injected samples from 5 to 100 μL at a concentration from 0.01 mM to 3 mM (Figure 6). Qualitative and quantitative analyses of the samples were carried out based on the parameters of the registered signal: the half-width, the retention time, the amplitude and the integral intensity (Figure 4D).

There is a clearly visible correlation between the size of the cations and the parameters of the electrical response of the nano detector. An increase of the ionic radius of the metal ion caused a decrease in the integral intensity and lower values of the half-width of the signal measured, in the same order (Figure 5A,B). Sodium ions (95 pm) can penetrate the monolayer more easily; therefore, they interacted with polyaniline nanofibrils more effectively. On the other hand, barium ions (135 pm) are less mobile (well-trapped), and their influence was much weaker.

The effectiveness of the interactions of specific metal ions trapped in a Si2t alkanethiol monolayer depended on the ionic radius, as a consequence of the effective electric field generated in vicinity of polyaniline nanofibers. The lowest values of the integral intensity of the signal for Ba^2+^ ions can be explained by the fact that these ions generated a less intensive effective electric field (E_ef_)—the charge +2 was shielded by a maximum number of electrons.

This can be described correctly (in accordance with the experiments) even through the simplest model. The effective electric field of the effective charge q_ef_ at a distance of r is described by E_ef_ = q_ef_/r^2^, and this influences the polyaniline nanofibrils, modifying the concentration of the charge carriers inside and the electrical conductivity (like a “gate” in an FET “channel”). 

Thus, the effect depends on the effective charge q_ef_ = Q/n_e_ and is expected to be a linear function of the ratio of Q/n_e_, where Q is the charge of a metal ion (in our case, Q = +1 or +2, for mono- and divalent ions respectively), and n_e_ is the number of electrons (n_e_ = Z-Q, where Z is the atomic number of a metal).

The conclusion was confirmed by the experiments. The integral intensity of the signals measured for all examined ions was linearly dependent on 1/n_e_ because of the influence of the electrons (shielding, Figure 5C). The effect depends on the effective electric field, which was different for ions of Q = +1 and Q = +2, as presented in Figure 5D. The shielding still dominated the influence of real charges +1 or +2, and one can clearly conclude that the device was much more sensitive to sodium ions than to any others examined. The conclusion is supported by measurements at different concentrations of ions.

The increase in the concentration of metal ions in the sample causes growth of integral intensity of the detector response (Figure 6) because of the increasing number of metal ions trapped in the alkanethiol monolayer. These influenced the polyaniline fibrils, which in turn led to discernible changes in the electrical conductivity.

The sensitivity of the new device to particular ions were evaluated by an analysis of the coefficient of linear regression (Figure 6). The highest value, 25.18, was obtained for the Na^+^ ion. In this case, the correlation coefficient was Rk = 0.993.

The coefficients of linear regression and the correlation coefficients (in brackets) for other ions were as follows: Ca^2+^ 22.12 (Rk = 0.981), Sr^2+^ 19 (Rk = 0.947), Ba^2+^ 12.41 (Rk = 0.971).

## 4. Discussion

The mechanism of the action of the new detector resembles a field-effect transistor (FET) [27,33]. The alkanethiol monolayer works as an insulator (Pnt component) between an FET “source” (S) or “drain” (D) electrodes and the “gate” (G), but also otherwise—because of presence of Si2t domains—it plays the role of an ion trap, owing to its appropriate structure. Metal ions are trapped in the vicinity of polyaniline nanofibers, resulting in a change of their electrical conductivity, owing to a field effect. That is why the device resembles an FET. The final result depended on an effective electric field intensity of a specific metal ion. This is a continuous-flow system; thus, the electrical response of the detector is dynamic, specific with respect to the intensity and shape of the signal, registered in a function of time. It exhibits a rapid response (about 20 s) and high sensitivity for small samples injected 5–100 μL. The detector, based on PANI nanofibrils, in combination with the functional monolayer (the composition of Pnt/Si2t in a quantitative ratio of 4:1) on the gold electrodes, exhibited the highest sensitivity to Na^+^ ions, and the lowest to Ba^2+^.

This can be explained by the easy penetration of the small cations into the complexing cavities. We observed that change in the concentration of metal ions from 0.01 to 0.09 mM did not significantly alter the integral signal intensity of the nanodetector. A clear increase in the integral intensity for all ions was recorded at concentrations above 0.1 mM. Thus, the practical detection limit of concentration was estimated to be about 0.1 mM for 75 μL of injected sample.

This is enough for many applications, and even now (at the level of “proof of concept”), the device could be useful for rapid detection of selected cations. For example, the most important electrolytes in the body include sodium (135–145 mM), potassium (3.5–5.1 mM), calcium (2.25–2.75 mM) and magnesium (0.65–1.2 mM). The indications for testing electrolytes in the serum include: cardiac arrhythmias (most importantly sudden cardiac arrest, SCA), abnormal blood pressure, headaches and muscle cramps, swelling of the lower extremities and the monitoring of water in the body.

In the system designed for practical use, a microfluidic-type device and the automatic, computer-aided acquisition and processing of the measured signal are applied (Appendix B).

## 5. Conclusions

This is a new concept, based on nanomaterials and nanotechnology. Polyaniline micro- and nanofibers and the thiol monomolecular layer have been successfully used as an active material in a new nanodetector of metal ions. The electric field of metal ions trapped in the vicinity of polyaniline nanofibrils was able to change the electrical conductivity of individual fibrils and the whole network. In a continuous-flow system, the electrical response of the nanodetector was almost linearly dependent on the concentration of the cations. It exhibited a fast response (about 20 s) and high sensitivity (with a practical detection limit of 0.1 mM, using a sample volume of 5–100 μL), sufficient for most applications. However, this is a “proof of concept”; the nanodetector is long-living, enabling multiple analyses with the same detecting unit. 

The results presented are very convincing and promising; thus, a microfluidic version of the new nanodetector has been designed for practical use, with automatic data processing supported by a computer. The advantage is instant response, easy operation and simple construction. The device is universal, of a long lifetime.

A microfluidic version of the device, supported by computer-aided processing of the measured signal, uses the open database for fast qualitative and quantitative automatic analyses of the metal ions. It was designed as an “intelligent” device for possible implementation and practical use. 

Owing to nanotechnology approach, the device is easy to fabricate with the use of organic nanomaterials, which is simple and inexpensive.

## Figures and Tables

**Figure A2:**
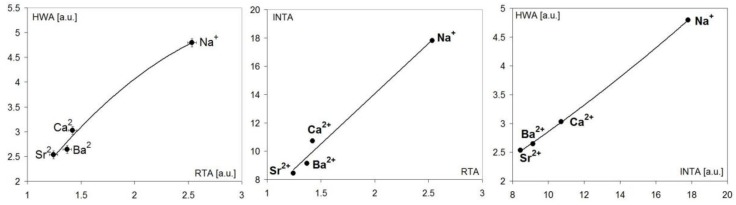
Correlations between HWA, RTA and INTA signal parameters for the tested ions illustrate a possibility of their automatic identification. These parameters are defined as HWA = HW/A, RTA = RT/A, INTA = INT/A, where A is the amplitude, RT—the retention time, HW—the half-width and INT—the integral intensity of the measured signal.

**Figure A3:**
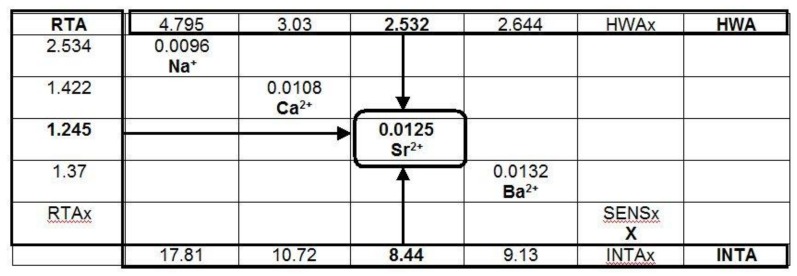
A schematic presentation of the automatic identification process. Parameters RTA, HWA and INTA are used to identify the metal ion. Sensitivity parameter SENS, defined as SENS = c[mM]/INT[a.u.], where c is the concentration of the metal ion [mM], and INT – the integral intensity of the signal. SENS is calculated during the calibration, together with other parameters, RTA, HWA and INTA, and used to evaluate the concentration of the analyzed metal ion with the aid of the integral intensity, INT, of the measured signal, e.g., for metal X, c_x_ = SENS_x_ × INT_x_.

## Appendix A

Preliminary systematic studies of blocking monolayers of different composition.

Monolayers that are deposited at the surface of a clean gold electrode and the current were measured. They were measured in water as flowing medium, when the sample of 75 μL of 0.15 M of NaCl was injected, i.e., at a concentration about 300 times higher than what is normally applied (0.5 mM), and thus, the measured current was also much higher. Polyaniline was not deposited in this case (Figure A1a); thus, the current is clearly ionic.

The tests are summarized in Figure A1b, together with a blank experiment, during which no modification of gold electrodes was applied. The most effective are: a monolayer formed of pentane-1-thiol Pnt (among one-component SAMs) and mixed thiols: pentane-1-thiol (Pnt), 3-[tris(2-methoxyethoxy)silyl]-propane-1-thiol (Si2t) at a molar ratio of 4:1 and, in the case of complex SAMs, Pnt-Si2t. The best quality and stability against 0.15 M of NaCl (the concentration of salt was about 300 times higher than what is normally applied, 0.5 mM), measured as the lowest value of ion current, was observed for a SAM of a complex structure, Pnt-Si2t (4:1), and this layer was then used as a basic matrix in further studies (Figure A1b and Figure 3A).

## Appendix B

In the system designed for practical use (the detailed technical information are to be patented), the automatic, computer-aided acquisition and processing of the measured signal were applied.

This enabled the instant calculation of characteristic parameters: HWA, RTA and INTA, defined as HWA = HW/A, RTA = RT/A, INTA = INT/A, where A is the amplitude, RT—the retention time, HW—the half-width and INT—the integral intensity of the measured signal (in the case of mixtures, if necessary, preceded by a signal deconvolution).

These parameters were used for automatic identification of ions by comparison with the database, which was first formed through the calibration procedure (Figure A2 and Figure A3). The quantitative information and the concentration of ions in the analyzed sample were proportional to the integral intensity of the signal (Figure 6), and they were calculated based on the sensitivity parameter of the system for each ion, which was defined during the calibration and stored in the database (Figure A3).

A direct analysis of complex mixtures was possible through calibration using the same or a similar complex mixture, in which the concentration of only one component was changed. This is a practical and effective procedure, which has successfully been tested with nanobiodetectors [31,32]. Advanced, computer-aided signal deconvolution to separate the information about the components is an alternative option.

To summarize, based on experimental data, RT, HW, INT and A, RTA, HWA and INTA signal parameters were calculated and then used to identify the metal ion, including the evaluation of the concentration in the analyzed sample. All with the aid of the database, e.g., when calculated parameters of the measured signal are RTA = 1.245, HWA = 2.532, INTA = 8.44, the result is Sr^2+^ (Figure A3). When the integral intensity of the measured signal (INT) reaches a value of 40, the concentration of Sr^2+^ in the analyzed sample is c = 40 × 0.0125 = 0.5 mM (because of the sensitivity of Sr^2+^, SENS = 0.0125, evaluated during the calibration and included in the database; Figure A3).

The database is practically unlimited, thus, the system is always open for new ions (including organic ones) to be analyzed, which depends only on the information in the database, collected during calibration. Direct analysis of complex mixtures is also possible. The procedure is analogous to the automatic identification of bacteria and spores with the use of a nanobiodetector, earlier developed in our laboratory [31,32].
